# Collaborative Annotation Redefines Gene Sets for Crucial Phytopathogens

**DOI:** 10.3389/fmicb.2019.02477

**Published:** 2019-11-07

**Authors:** Helder Pedro, Andrew D. Yates, Paul J. Kersey, Nishadi H. De Silva

**Affiliations:** European Molecular Biology Laboratory, European Bioinformatics Institute, Hinxton, United Kingdom

**Keywords:** community gene annotation, apollo, Ensembl genomes, fungi, plant pathogens

## Abstract

Accurate and comprehensive annotation of genomic sequences underpins advances in managing plant disease. However, important plant pathogens still have incomplete and inconsistent gene sets and lack dedicated funding or teams to improve this annotation. This paper describes a collaborative approach to gene curation to address this shortcoming. In the first instance, over 40 members of the *Botrytis cinerea* community from eight countries, with training and infrastructural support from Ensembl Fungi, used the gene editing tool Apollo to systematically review the *entire* gene set (11,707 protein coding genes) in 6–7 months. This has subsequently been checked and disseminated. Following this, a similar project for another pathogen, *Blumeria graminis* f. sp. *hordei*, also led to a completely redefined gene set. Currently, we are working with the *Zymoseptoria tritici* community to enable them to achieve the same. While the tangible outcome of these projects is improved gene sets, it is apparent that the inherent agreement and ownership of a single gene set by research teams as they undergo this curation process are consequential to the acceleration of research in the field. With the generation of large data sets increasingly affordable, there is value in unifying *both* the divergent data sets and their associated research teams, pooling time, expertise, and resources. Community-driven annotation efforts can pave the way for a new kind of collaboration among pathogen research communities to generate well-annotated reference data sets, beneficial not just for the genome being examined but for related species and the refinement of automatic gene prediction tools.

## Introduction

Plant pathogens continue to threaten food security and impose a significant economic burden. A deeper understanding of gene function in both host and pathogen is critical in identifying strategies to detect, prevent, and manage these diseases. There are, however, gaps in our knowledge for crucial pathogens, and even when their genomes have been sequenced, discrepant gene sets circulate among different research groups owing to preferences in tools and protocols.

With easy access to affordable technologies, sequencing now forms a routine component in many projects, leading to burgeoning volumes of data and an increasing demand for tools to give that data meaning. To that end, genomic sequences typically undergo automatic gene annotation as a first step. However, despite advances in gene prediction algorithms, they still cannot automatically resolve all complexities surrounding the precise location and structure of genomic elements. It is not uncommon for there to be missed genes, false predictions, and incorrectly merged or split gene models in a published gene set. Functional predictions can also be made using computational algorithms; again, these are useful but can be prone to mis-identification (Stein, 2001; McDonnell et al., 2018) and reliant on the accuracy of the gene prediction. While automation is necessary to provide a scalable approach to annotation, some manual intervention is instrumental to increase accuracy, identify errors and peculiarities, and potentially improve automated gene prediction tools by using these observations. Manual curation generates well-annotated reference genomes that are vital to almost all processes of automatic annotation that rely heavily on propagating reliable data from a reference to related genes/species. Reference knowledge is patchy, as research and interest are naturally focused on specific biological problems within a given portion of the taxonomy. However, where a research community does exist, we can harvest its knowledge and propagate that information to taxonomically adjacent species.

There are several approaches to manual gene curation (Loveland et al., 2012) ranging from dedicated teams examining gene structures to individual data owners with permission to edit specific genes (Grigoriev et al., 2014) to open Wikipedia-style community editing. However, manual gene curation is a significant undertaking and unfunded in all except key model species. It requires time, expertise, and supporting data; all three of which may not be available in abundance within a single research team. It is likely that different research groups generate transcriptomic and other data to supplement their research objectives, which, if combined, could provide tremendous insight into gene structure and location. However, these groups may be geographically dispersed, low on time and resources to make this happen, and without all the skills (e.g., software development, systems administration, and gene model curation) needed to interpret these data and effect changes in gene models.

We believe that a collaborative approach can provide a solution to this problem. We present here our efforts to marshal technology, data, and person power to support collaborative gene curation during the PhytoPath project (Pedro et al., 2016), the challenges faced, and the lessons learnt.

## Marshaling Technology, Data, and Person Power to Expedite Manual Gene Annotation

**Figure 1:**
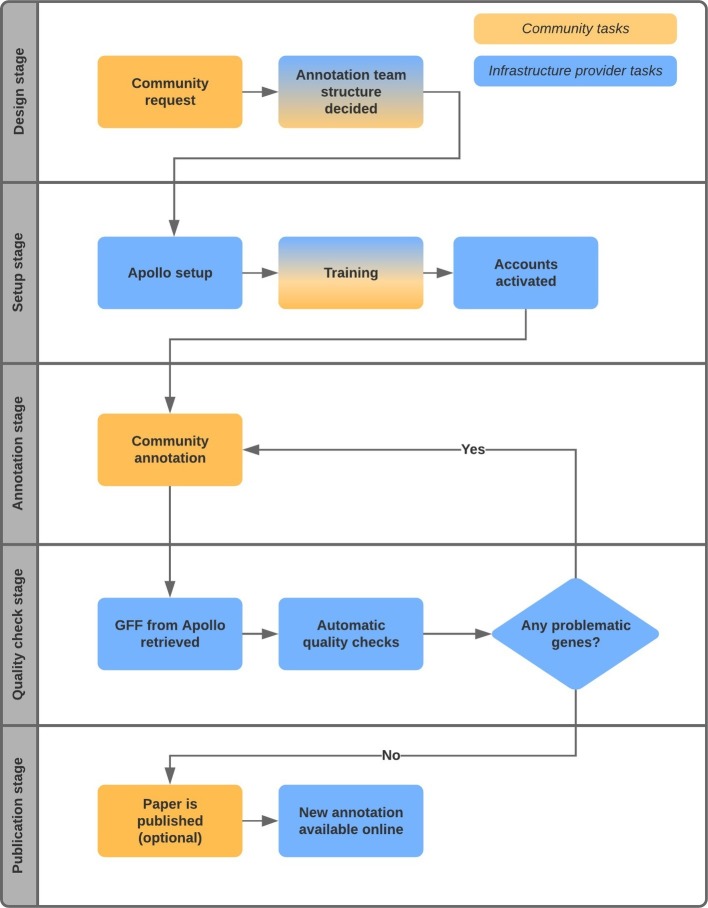
Workflow of collaborative gene curation projects.

Research groups can collaborate to redefine the gene set for their species of interest by pooling together additional data and gene predictions of related species. Figure 1 illustrates the typical workflow of such a project. The process usually begins with an interested community, often spearheaded by an enthusiast in the research area, approaching a resource like Ensembl Fungi (RRID:SCR_008681; Kersey et al., 2018) that can act as a hub and infrastructure provider. In the examples that follow, Ensembl Fungi also sets up an instance of Apollo [RRID:SCR_001936] and hosts the associated databases and infrastructure.

There is of course a multitude of other gene editing software used by curation teams [for example, Artemis (Carver et al., 2012) and ZMap (Madupu et al., 2010)]. The advantages of Apollo include easy customization, installation, and use, which are especially important for volunteer curators whose main work is elsewhere. Apollo is a plugin for the JBrowse genome viewer allowing geographically dispersed participants to edit gene models collaboratively, view history and concurrent changes, and, in later versions, even annotate variants on the sequence (Dunn et al., 2019) with just an internet browser. This has made Apollo an extremely attractive option for many projects on diverse species. For instance, VectorBase (Giraldo-Calderón, 2015) uses Apollo in an open-ended curation effort to annotate vector genomes; the Genome Decoders project [a collaboration between WormBase (Lee et al., 2018) and the Institute for Research in Schools^1^] uses Apollo for the annotation of a parasitic worm genome by a cohort of around 1,000 students; and other projects have used Apollo successfully to annotate the genomes of fungal pathogens (Syme et al., 2016).

Typically, some of the participating groups in the annotation project will have data that can be loaded as evidence *tracks* into Apollo. This is usually short read transcriptomic data but may also include PacBio Iso-Seq data and protein sequences. Alignments to data derived from related species (including not only primary sequence data but also previously identified gene models, homology, and conservation data) may also be shown.

The infrastructure provider (in this case, Ensembl Fungi) then conducts a training webinar in which participants can connect to live, ask questions, and share experiences. We create user accounts on the Apollo instance, manage permissions, and provide general technical support to the curators. The work can be distributed too, typically by generating gene lists and chromosome regions or known problem regions for each participant to examine.

At this point, the curation work begins. Based on the evidence visible in Apollo, most of the changes made by the participants are to the structure of the gene models. Some types of structural changes are adjusting the boundaries of transcripts and untranslated regions (UTRs), recording new (biologically meaningful) splice variants, choosing the best from multiple predicted gene models, removing incorrect gene predictions, and merging gene fragments.

In some cases, functional annotation can be enhanced too, based on *a priori* experience of a particular gene family or related species. This can involve assigning new names or functions to the gene [for example, by adding Gene Ontology (GO) terms or changing the type of the gene].

This process can take several months, with peaks and troughs in activity according to the other workloads of the participants. Once complete, the resulting annotations are extracted as GFF files from Apollo and put through a set of automatic quality checks; for instance, detecting internal stop codons, mismatches in coordinates and strand, and duplicated gene names that may have missed the curators’ checks. Once fixed (with feedback from the participants), the data are disseminated. Due to the data submission protocols in the public archives such as the European Nucleotide Archive (ENA) or GenBank, it has not been straightforward for non-data-owners to submit the updates to these repositories. However, in the examples presented, the data were quickly integrated into Ensembl Fungi, compared with predictions from other genomes and used to construct gene trees and determine homology, and disseminated to the scientific community within the context of additional data types (e.g., variation data). In two of the case studies discussed below, the work has also been included in journal publications.

### Case Studies

Our first project included 42 members of the *Botrytis cinerea* community from 10 institutions in eight countries. The project considered one initial gene set and three tracks of additional data (alignments to proteins of closely related species and two tracks displaying RNA-Seq reads). The group were able to check the entire gene set in 6–7 months, editing 11,612 genes and adding 35 new genes. The ensuing gene set was integrated into Ensembl Fungi and released *via* our usual web browser and programmatic APIs and was also included in a publication (van Kan et al., 2017). A similar project for *Blumeria graminis* f. sp. *hordei* also concluded within 6–7 months but only involved four members of the *B. graminis* community from two institutions and countries. As with the previous genome, there was one initial gene set but 20 tracks of evidence. The final gene set contained 5,541 genes from the original set, 1,943 deleted genes, and 1,577 new genes. This too was embedded within Ensembl Fungi and a publication (Frantzeskakis et al., 2018). We are currently engaged in a community annotation project around the important wheat pathogen *Zymoseptoria tritici* involving 26 species experts from eight institutions in four countries. *Z. tritici* currently has five different gene sets. The Apollo instance for *Z. tritici* displays all five, along with 40 evidence tracks. Genes in *Z. tritici* are densely packed; the use of new RNA-Seq data from different groups has greatly helped disambiguate neighboring genes that had wrongly been merged in the automatic predictions.

## Lessons Learnt for Future Collaborative Annotation Projects

As with other collaborative projects, we believe that there are three key aspects that communities wanting to embark on such annotation projects should think about: forming the community, keeping the momentum up, and quality control of annotation.

### Forming the Community

As observed by our case studies, the size of the community involved can vary significantly (4–42, in our examples) and still have similar levels of impact. This is hugely encouraging. When we examined the gene edit logs in Apollo for the *B. cinerea* and *B. graminis* projects, we noticed that just a few annotators (two or three, and in both cases including the project leader) can end up doing over half the total gene edits, while the rest of the edits were equally shared by the other annotators. In the *Z. tritici* project, where we distributed lists of genes that needed checking at the beginning, the edits done so far have been evenly spread among the active participants. The most pressing concern for volunteers in these projects is often time. Since the Apollo instance is always online, curators can log in whenever they have a spare morning/afternoon; some changes require just a few seconds (to confirm gene models are correct), and others need up to 20 minutes to change (UTR boundaries and other structural alterations).

Often the greatest determinant of success is an enthusiastic champion (leader) who drums up support from her/his collaborators and fuses the community expertise and resources. The groups generating the supporting evidence are doing so for other research purposes, and the fact that it can also be used to improve the gene set is a beneficial side effect to their main project. The “leader” also oversees the project and acts as a liaison between new members wanting to join, the infrastructure provider, and existing annotators. During the projects discussed, we advertised *via* various forums (e.g., twitter, Ensembl homepages and blogs^2^) inviting interested members of the research community to get in touch. We have had a variety of responses to these outreach efforts, from thank you emails from strawberry growers to scientists wanting to join the annotation effort.

In addition to the broad benefits of having a better gene set, more could be done to incentivize members to join these projects and make their time spent annotating worthwhile. In particular, a mechanism is needed by which all participants in a project can be given due credit for their contributions, perhaps by including a list of participant names alongside the resultant gene set or a fair accountability system for gene edits (similar to edit histories in Wikipedia and git). We have done the former for the *Botrytis cinerea* project by including a list of participants on the species homepage in Ensembl Fungi. The latter is more complex and needs further thought and clarification around issues such as the types of edits that get acknowledged and which members in the audit trail of a gene get mentioned.

### Keeping the Momentum Up

After initial set up, there is the task of keeping the momentum and morale up. The participants bring their own experience and strengths into this effort. We found that the training webinar at the start greatly helped kick-start the process, along with a clear set of starting tasks (a list of genes or regions assigned to each curator, for example) and engagement by the community “leader.” As annotation progresses, we believe a regular check-in or group call will greatly assist in keeping the pace up and raising awareness of problems and conflicts in annotations. Some groups are pushed further by internal deadlines such as paper submission dates or needing to get the data into the public forum within a particular time frame.

### Quality Control of the Annotation

The third challenge is standardizing the curation. The collective expertise within a group may be extensive but diverse. The participants are typically bioinformaticians, biologists, PhD students, or postdoctoral researchers with background knowledge relevant to the species. There is trust that the curators will apply their biological knowledge to assess gene structures effectively (in contrast to an open model where anyone can edit genes but then later needs review by an expert). In cases where there are multiple gene sets, the curator’s job may simply be to select the best predicted gene model for the given loci. In our opinion, this is a straightforward decision in most cases when viewed alongside the evidence tracks. However, in cases where the structure of the gene needs alteration (adding or removing exons or dragging the ends of UTRs for instance), it is difficult to guarantee that different participants will apply the same rules to do this or, in fact, that the same participant will do the *exact* same thing each time she/he annotates.

The first way of addressing this is through the initial training webinar, by laying out clear rules and guidelines. We have also developed a tutorial (available freely) incorporating general rules applicable to gene annotation and a growing collection of species-specific quirks that need to be kept in mind for particular pathogens as reported by curators in our projects^3^. This material is also incorporated in the annual Wellcome Advanced course on fungal pathogens and distributed to the participants to be used as needed in their own research institutions and countries. The second approach (used by the *Zymoseptoria tritici* community) is to select a small subset of genes and to ask a group of experienced curators to evaluate if the decisions taken in each case are uniform and sensible. Comments regarding consensus or disagreements can be recorded, reported back to the curation team, and used to edit the tutorial and guidelines. This exercise can be repeated periodically. Any conflicts that arise during the annotation process have typically been resolved by raising it with the larger group during meetings or by email, or discussion with the group leader.

The third way to address this is by automated checks and controls. Apollo already does not allow (or makes extremely difficult) non-standard positioning of exons and other genomic elements, automatically calculating the nearest upstream or downstream splice junction, for example. Furthermore, the automatic quality checking done by Ensembl Fungi can also filter gross errors in gene annotation (for example, detecting internal stop codons, mismatches in coordinates and strand, and duplicated gene names) but is insensitive to minor inconsistencies between changes. In combination, we believe these approaches have been effective in checking the changes made to the genes in our projects.

A fourth, more labor intensive, method is asking multiple reviewers to check each region. With the ability to view annotation history in Apollo, members can review the decisions taken previously and assess the gene model against the evidence.

## Discussion

We believe our approach provides a manageable solution for handling the mammoth task of manual gene annotation in the absence of dedicated funds or teams. Pooling the expertise, resources, and time of willing communities enables a wide range of geographically distant members to participate in a common process, to share, and to validate the identification of contradictions and the misrepresentation of data on the genomes (for example, non-canonical splice sites). Thus corrected, the data sets emerging from these projects can be used to improve the gene sets for closely related genomes and downstream analysis (for example, the development of pangenomes and extrapolating function to orthologous genes). For instance, the *Botrytis cinerea* gene set discussed above has already been used to evaluate the accuracy of automated gene predictions in eight other *Botrytis* genomes (personal communication by Jan van Kan). Every new gene predicted was aligned to genes in the manually verified gene set, and based on sequence similarity and protein length, it was either passed or marked as dubious (requiring a manual check against transcriptomic evidence and homology to other fungal genomes). Manually verified gene sets can play a vital role in the emerging landscape of ambitious large-scale sequencing projects, training gene prediction algorithms, and neural networks to produce and recognize correct annotation for many other species.

The dialogue and collaboration between community members (who may previously have been using divergent gene sets) have enormous impact. The end result of an entire community agreeing on and taking ownership of a single gene set is a major stepping stone to accelerating the development of the field. We are excited by the prospect of this approach becoming commonplace and will continue to engage in community-driven curation efforts.

## Data Availability Statement

Publicly available datasets were analyzed in this study. These data can be found here: GCA_000143535.4, GCA_000151065.2, and GCA_000219625.1.

## Author Contributions

HP facilitated the community annotation projects from 2010 to 2017 and contributed to the paper. ND is now responsible for these projects and wrote the paper. AY has overall responsibility for the microbial portals within Ensembl and revised the manuscript. PK led the BBSRC-funded PhytoPath project and revised the manuscript. All authors read and approved the manuscript.

### Conflict of Interest

The authors declare that the research was conducted in the absence of any commercial or financial relationships that could be construed as a potential conflict of interest.

## Footnotes

^1^http://www.researchinschools.org/

^2^http://www.septoria-tritici-blotch.net

^3^http://ftp.ebi.ac.uk/pub/databases/ensembl/training/2019/FungalPathogens/Community_gene_annotation_using_Apollo_WTAC.pdf
